# Dynamics of Immune Reconstitution and Activation Markers in HIV+ Treatment-Naïve Patients Treated with Raltegravir, Tenofovir Disoproxil Fumarate and Emtricitabine

**DOI:** 10.1371/journal.pone.0083514

**Published:** 2013-12-18

**Authors:** Nicholas T. Funderburg, Adriana Andrade, Ellen S. Chan, Susan L. Rosenkranz, Darlene Lu, Brian Clagett, Heather A. Pilch-Cooper, Benigno Rodriguez, Judith Feinberg, Eric Daar, John Mellors, Daniel Kuritzkes, Jeffrey M. Jacobson, Michael M. Lederman

**Affiliations:** 1 Case Western Reserve University, Division of Infectious Diseases and HIV Medicine, Cleveland, Ohio, United States of America; 2 The Johns Hopkins University, Baltimore, Maryland, United States of America; 3 Center for Biostatistics in AIDS Research, Harvard School of Public Health, Boston, Massachusetts, United States of America; 4 University of Cincinnati Medical Center, Cincinnati, Ohio, United States of America; 5 Los Angeles Biomedical Research Institute at Harbor UCLA Medical Center, Torrance, California, United States of America; 6 University of Pittsburg Medical Center, Pittsburg, Pennsylvania, United States of America; 7 Brigham and Women’s Hospital and Harvard Medical School, Boston, Massachusetts, United States of America; 8 Drexel University College of Medicine, Philadelphia, Pennsylvania, United States of America; Asociacion Civil Impacta Salud y Educacion, Peru

## Abstract

**Background:**

The dynamics of CD4+ T cell reconstitution and changes in immune activation and inflammation in HIV-1 disease following initiation of antiretroviral therapy (ART) are incompletely defined and their underlying mechanisms poorly understood.

**Methods:**

Thirty-nine treatment-naïve patients were treated with raltegravir, tenofovir DF and emtricitabine. Immunologic and inflammatory indices were examined in persons with sustained virologic control during 48 weeks of therapy.

**Results:**

Initiation of ART increased CD4+ T cell numbers and decreased activation and cell cycle entry among CD4+ and CD8+ T cell subsets, and attenuated markers of coagulation (D-dimer levels) and inflammation (IL-6 and TNFr1). These indices decayed at different rates and almost all remained elevated above levels measured in HIV-seronegatives through 48 weeks of viral control. Greater first and second phase CD4+ T cell restoration was related to lower T cell activation and cell cycling at baseline, to their decay with treatment, and to baseline levels of selected inflammatory indices, but less so to their changes on therapy.

**Conclusions:**

ART initiation results in dynamic changes in viral replication, T cell restoration, and indices of immune activation, inflammation, and coagulation. These findings suggest that determinants of T cell activation/cycling and inflammation/coagulation may have distinguishable impact on immune homeostasis.

**Trial Registration:**

Clinicaltrials.gov NCT00660972

## Introduction

HIV-1 infection is characterized by T cell activation, inflammation, and hyper-coagulation, yet, effects of antiretroviral therapy (ART) on dynamics of these indices and correlates of CD4+ T cell reconstitution are incompletely understood. Depletion of CD4+ T cells is driven in part by HIV-1 replication [1]; however, increasing evidence implicates immune activation as an important correlate, and possible determinant of CD4+T cell depletion and disease outcome [2–4]. Potential drivers of activation include: activation by components of HIV-1 [5–7], by copathogens such as cytomegalovirus [8], by Toll-like receptor (TLR) ligands translocated from the damaged gut [9–12], or by homeostatic responses to lymphocytopenia [13,14]. Exposure of peripheral blood mononuclear cells to TLR ligands activates T cells in vitro [6,15] and certain TLR ligands can drive memory CD4+ T cells into cell cycle and death [15]. As ART results in decreased plasma levels of HIV-1 and both plasma inflammatory markers and cellular markers of T cell activation [9,10,16–18], we hypothesized that a detailed assessment of the decay dynamics of these indices after ART initiation would provide insight into the determinants of immune restoration in treated HIV-1 infection; and, by inference, into the determinants of cellular turnover, inflammation, and immune deficiency in HIV-1 disease. 

## Materials and Methods

### Ethics Statement

This study was approved by institutional review boards at all participating sites: Brigham and Women's Hospital Clinical Research Site (CRS), Johns Hopkins Adult AIDS CRS, UCSD, AVRC CRS, University of Rochester ACTG CRS, AIDS Care CRS, Washington University CRS, The Ohio State University AIDS CRS, MetroHealth CRS, Northwestern University CRS, The Miriam Hospital ACTG CRS, Vanderbilt Therapeutics CRS, IHV Baltimore Treatment CRS, University of Colorado Hospital CRS, Houston AIDS Research Team CRS, and the Harlem ACTG CRS. This trial is registered with Clinicaltrials.gov # NCT00660972. 

The protocol for this trial and supporting CONSORT checklist are available as supporting information; see Checklist S1 and Protocol S1.

### Study Design

A5248 was a prospective, open-label, multicenter, pilot study performed in the United States of America between June 2008 and April 2010. HIV-1 infected ART-naïve patients with plasma HIV-1 RNA levels >10,000 and <300,000 copies/mL and any CD4+ T cell counts enrolled after providing written informed consent. Subjects were ineligible if screening genotype identified major nucleoside reverse transcriptase inhibitor, non-nucleoside reverse transcriptase inhibitor, or protease inhibitor resistance mutations. HIV-1 RNA and CD4+ T cell count were measured at pre-entry and entry visits and the geometric and arithmetic means were used, respectively, to establish baseline values. Participants were treated with raltegravir (RAL, 400 mg twice daily) and emtricitabine/tenofovir disproxil fumarate (FTC/TDF, 200mg/300mg once daily). Data obtained after virologic failure (VF) or clinical rebound (CR) on or before week 48 were excluded from analyses. Virologic failure was defined as a confirmed plasma HIV-1 RNA level ≥1000 copies/mL at or after 16 weeks and before 24 weeks, or ≥200 copies/mL at or after 24 weeks. Clinical rebound was defined as confirmed plasma HIV-1 RNA >0.3 log_10_ c/mL above the previous measurement [19] . 

### Sample collection

Blood samples were collected at pre-entry, entry, day 2, day 7, and weeks 2, 4, 8, 24, and 48. Absolute CD4+ and CD8+ T cell counts were obtained in real-time. Peripheral blood mononuclear cell (PBMC) samples were cryopreserved until analyzed in batch. Whole blood samples collected in EDTA-containing tubes were centrifuged for 10 minutes at 495 x g and plasma was frozen at -80°C until thawed once and analyzed in batch. 

Plasma was assayed for HIV-1 RNA using the AMPLICOR HIV-1 Monitor version 1.5, UltraSensitive (US) protocol (≤50 copies/mL; Roche Molecular Systems, Branchburg, New Jersey, USA)

### Flow cytometry

CD4+ and CD8+ T cells were identified by size, granularity, and staining with antibodies to CD4 or CD8. The following antibody-fluorochrome conjugates (and appropriate isotype controls) were used: anti-CD4 (Pacific Blue, Becton Dickinson (BD) Pharmingen, San Diego, CA), anti-CD8 (Peridinin-chlorophyll-protein Complex, PerCP), Franklin Lakes, NJ), anti-CD45RA (allophycocyanin, APC,BD Pharmingen), anti-Ki-67 (phycoerythrin, PE, BD Pharmingen), anti HLA-DR (fluorescein isothiocyanate, FITC (BD Biosciences), anti CD38 (PE, BD Biosciences), and anti-CCR7 (PE-Cy7,BD Pharmingen). To assure accuracy when analyzing lymphocyte subsets, only quadrants with more than 300 cells were reported. For analysis of intracellular Ki-67, cells were incubated with FACS Permeabilizing Solution (BD Biosciences) for 15 minutes, washed then stained with anti-Ki-67 antibody or with an isotype control antibody for 45 minutes in the dark.  Cells were then washed and fixed with 1% formaldehyde, and analyzed using an LSR II flow cytometer (BD). 

### Plasma assays

Levels of soluble CD14 (sCD14), tumor necrosis factor receptor type 1 (TNFr1), and interleukin-6 (IL-6) were measured using the Quantikine ELISA kits (all from R&D Systems Minneapolis MN). Levels of D-dimers were measured using the Asserachrom D-DI immunoassay (Diagnostica Stago, Asnieres France). 

Plasma levels of LPS were quantified using the Limulus Amebocyte Lysate (LAL) assay (QCL-1000, Lonza, Walkersville, MD) according to the manufacturer’s protocol as previously described [20]

### Control Samples

To compare immunologic indices to those found in health, cryopreserved PBMC and plasma samples from 21 healthy, HIV-uninfected control subjects were thawed and then cellular and plasma markers of immune activation were measured as above by the same laboratory. The inflammatory and coagulation indices data were reported previously [21]. The 21 healthy control donors included 11 men; their ages ranged from 23-60 years. 

### Statistical Methods

P-values were two-sided and not adjusted for multiple comparisons. For this exploratory analysis, nominal significance was attached to p-values of <5%. Wilcoxon signed rank tests were used to evaluate significance of changes from baseline. Wilcoxon rank sum tests were used to evaluate differences between findings in patients and controls. Spearman (rank-based) correlations were used to evaluate associations between outcomes

## Results

Thirty-nine subjects met criteria for immunologic evaluation. In subjects who exhibited VF or CR at any time on or before week 48, data are excluded after the earliest such event. Two subjects did not have viable PBMC available, but had plasma samples for analysis. Thus, sample sizes ranged from 37 (39 for soluble markers) at baseline to 27 at week 48. Baseline characteristics for the 39 study patients and 21 healthy controls are shown in Table 1. With administration of ART, plasma HIV-1 RNA levels fell rapidly and decreased significantly by day 2 (not shown). By day 28, median levels were < 50 copies/mL. 

**Table 1:** Baseline demographics and clinical characteristics of patients enrolled in A5248 and uninfected controls.

**Characteristic**	**HIV-1 Infected Patients(N=39)**	**HIV-1 Uninfected Controls**
		**(N=21)**
**Gender**	Male= 35 (90%)	Males = 11 (52%)
	Female= 4 (10%)	Females = 10 (48%)
**Age** (years)	Median = 44	Median= 37
	Range = 23-58	Range = 23-60
**Race/Ethnicity**		
White Non-Hispanic	18 (46%)	16 (76%)
Black Non-Hispanic	11 (28%)	2 (9%)
Hispanic (Regardless of Race)	7 (18%)	1 (5%)
Asian		1 (5%)
More than 1 Race	2 (5%)	
Unknown/Missing	1 (3%)	1 (5%)
**Plasma HIV-1 RNA** (c/mL)	Median = 37, 490	Not applicable
	Range= 6,644 - 619, 795	
**CD4+ T cell count** (cells/µL)	Median = 259	Median = 907
	Range= 150-599	Range= 544-1957

**Figure 1:**
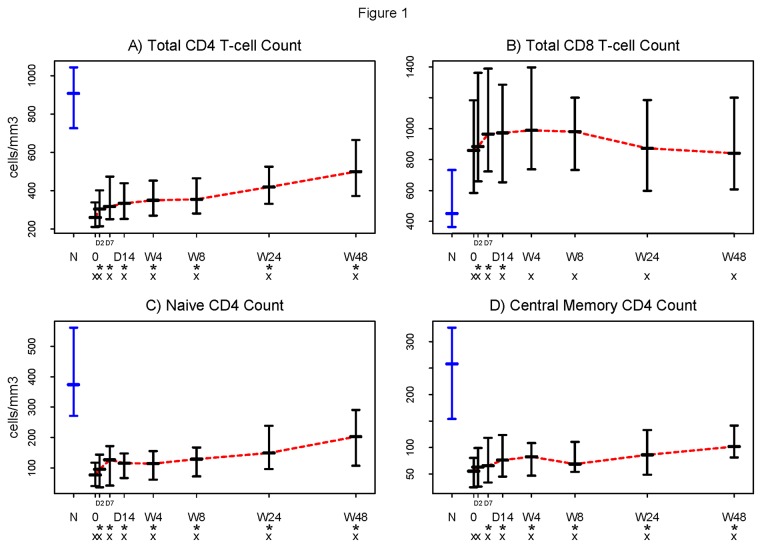
Initiation of antiretroviral therapy (ART) results in an increase in the number of CD4+T cells and a decrease in the number of CD8+ T cells. Absolute CD4+ and CD8+ T cell counts were obtained in real time on fresh whole blood samples. Lymphocytes were identified by flow cytometry based on size and granularity; T cell subsets were identified by positive expression of CD4 or CD8. The numbers of circulating A) CD4+ and B) CD8+ T cells within this HIV-1 infected patient population changed significantly from baseline by day 2 (p<0.001 and p<0.002, respectively), but did not reach levels seen in healthy controls within 48 weeks of ART treatment. C) naïve (CD45RA+ CCR7+) and D) central memory (CM, CD45RA-CCR7+) CD4+ T cell numbers increased significantly by day 2 (p<0.001) and day 7 (p=0.001) respectively. Symbols used in the figure: N = Normal controls (in blue). 0 = Baseline. D = Day (The two tick-marks between “0” and “D14” are Day 2 and Day 7). W = Week. * = Change from baseline significantly different from 0 (Wilcoxon signed rank p ≤0.05) . x = Significant difference from the normal controls (Wilcoxon rank sum p ≤0.05). - Horizontal bars represent 25^th^ (Q1), 50^th^ (Median), and 75^th^ percentiles. … Dotted line (in red) connects the medians over time.

With initiation of ART, circulating CD4+ T cell numbers typically increase biphasically, with a rapid first phase increase attributed to cellular redistribution from lymphoid tissues, and a second phase increase that has been attributed to homeostatic T cell expansion [13,14]. Here, we observed rapid first phase CD4+ T cell restoration reaching inflection around week 4, followed by a slower second phase restoration (Figure 1A), although CD4+T-cell counts through week 48 did not reach levels seen in uninfected controls. By week 48, however, 16 of 31 (52%) subjects had CD4+ T-cell counts exceeding 500 cells/uL. Circulating CD8+ T lymphocyte numbers also typically rise during first phase restoration, then may increase or diminish [16,22]. Circulating CD8+ T cell numbers rose transiently by day 2; but, by week 4, were no longer different from baseline levels and remained higher than levels seen among uninfected controls (Figure 1B). 

Naïve CD4+ T cells represent the broad repertoire of potential responses to neoantigens, while central memory (CM) T cells reflect anamnestic potential upon antigen re-exposure. In clinical studies, naïve CD4+ T cell numbers are predictive of total CD4+ T cell restoration [23], whereas CM CD4+ T cell turnover has been linked to disease progression in non-human primate models [24]. Naïve and CM CD4+ T cells increased significantly from baseline by days 2 and 7, respectively, (p<0.001 and p=0.001, Figures 1C and 1D), yet neither population reached levels seen in healthy controls even by week 48 (p<0.001 for both at week 48.) 

**Figure 2:**
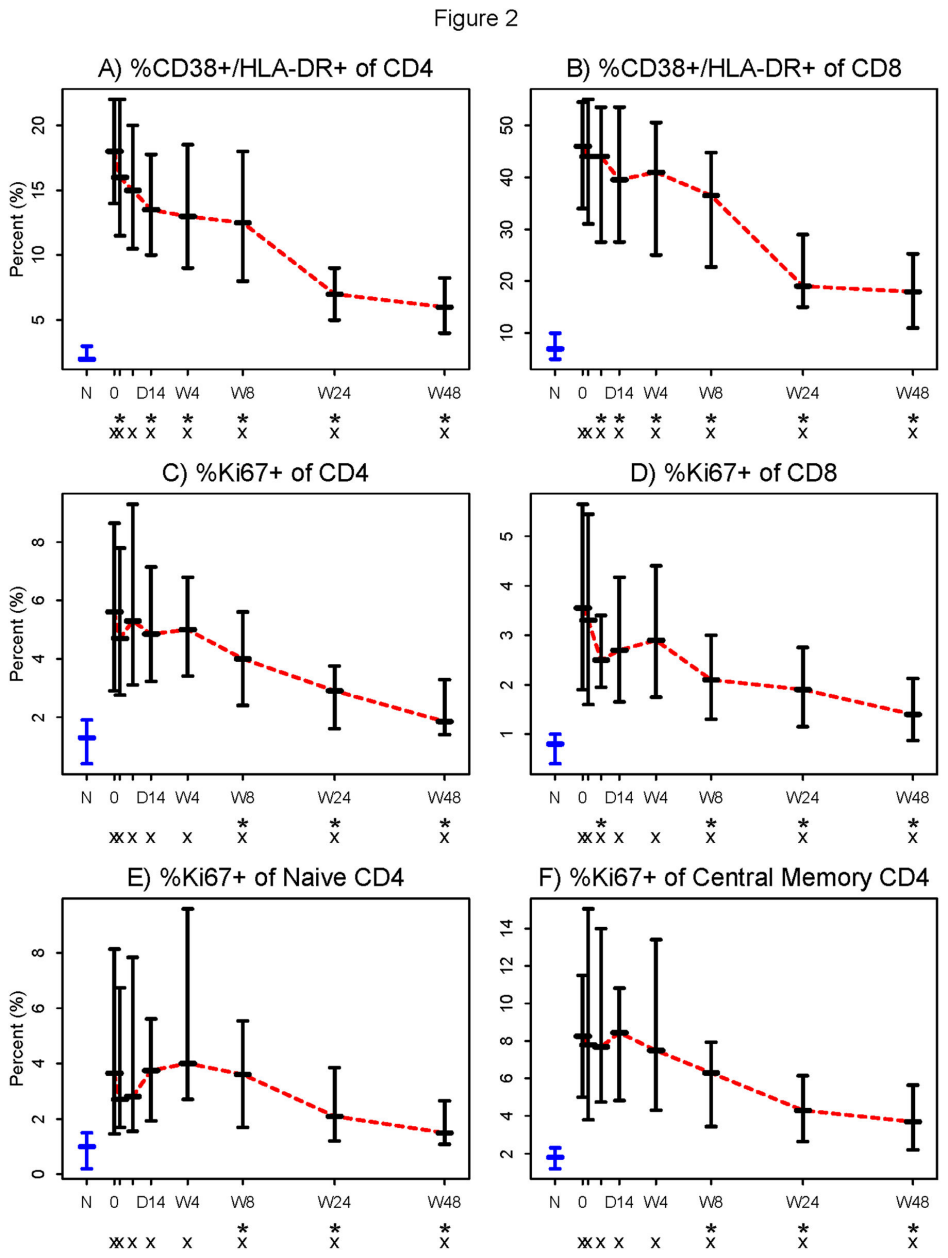
Proportions of activated (CD38+HLA-DR+) and cycling (Ki67+) CD4+ and CD8+ T cells decreased following initiation of ART. Among both A) CD4+ and B) CD8+ T cell populations, initiation of raltegravir plus emtricitabine/tenofovir resulted in a significant decrease from baseline in the proportion of activated cells by 2 days and 7 days (p= 0.05 and 0.015, respectively). By week 48, the proportions of activated CD4+ and CD8+ T cells in these patients did not approach the proportions measured in healthy controls. Also among both C) CD4+ and D) CD8+ T cell populations, initiation of ART significantly decreased the proportions of Ki67+ cells by week 8 and by day 7 respectively (p<0.001 and p=0.028). E) Naïve CD4+ T cells (CD4+,CD45RA+, CCR7+) and F) CM CD4+ T cells (CD4+,CD45RA-, CCR7+) that express Ki67 were significantly reduced from baseline by week 8 (p=0.005 and p=0.001, respectively). In all four T cell subsets, proportions of Ki67+ cells did not approach the proportions seen in healthy controls by week 48 of the study. Symbols used in the figure: N = Normal controls (in blue). 0 = Baseline. D = Day (The two tick-marks between “0” and “D14” are Day 2 and Day 7). W = Week. * = Change from baseline significantly different from 0 (Wilcoxon signed rank p ≤0.05) . x = Significant difference from the normal controls (Wilcoxon rank sum p ≤0.05). - Horizontal bars represent 25^th^ (Q1), 50^th^ (Median), and 75^th^ percentiles. … Dotted line (in red) connects the medians over time.

Expression of CD38 and HLA-DR has been directly linked to T cell depletion, and has been inversely related to T cell reconstitution on ART [2,3]. Here, proportions of CD38+/DR+ CD4+ and CD8+ T cells decreased significantly from baseline by 2 days and 7 days after initiation of therapy (p= 0.05 and 0.015, respectively, Figures 2A and 2B). At no time over 48 weeks did proportions of activated CD4+ and CD8+ T cells normalize to levels seen in healthy controls (p<0.001 for both).

Though these phenotypic indices of activation predict outcome in untreated HIV-1 infection, it is likely that T cell turnover is more closely linked to death of CD4+ T cells [24–26]. Here, the frequency of cycling CD4+ T cells did not fall significantly until week 8 (p<0.001, Figure 2C), but the increased proportions of cycling (Ki67+) CD8+ T cells fell rapidly from baseline by day 7 (p=0.028, Figure 2D) . Both remained elevated throughout the study when compared to levels in healthy controls (p<0.001 for both). The proportions of naïve and CM CD4+ T cells expressing Ki67 also did not decrease significantly until week 8 (p<0.001, Figure 2E, 2F), and their frequencies did not reach the proportions seen in healthy controls at any time. 

**Figure 3:**
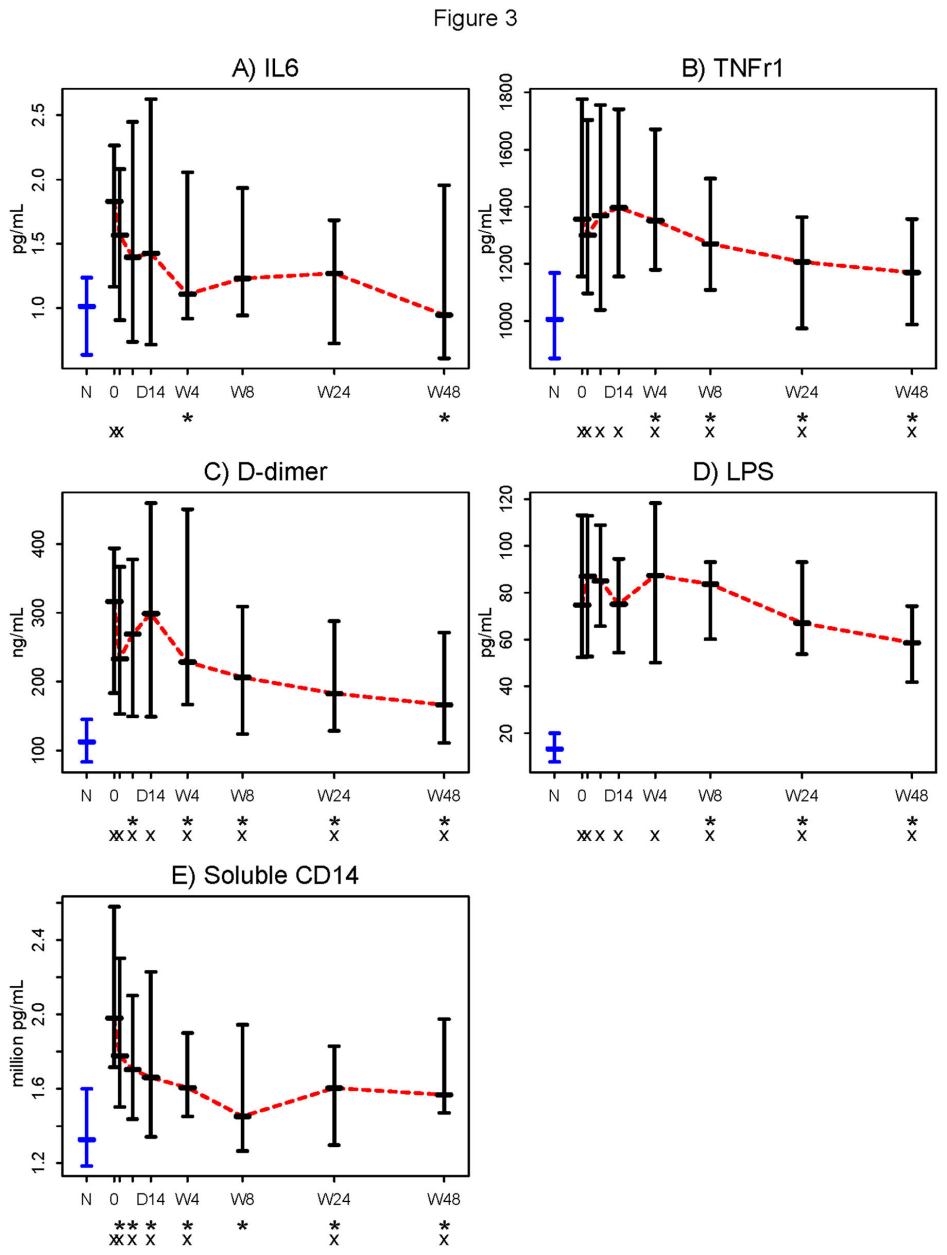
Markers of inflammation and coagulation are reduced following initiation of ART. Plasma samples were thawed and levels of A) interleukin-6 (IL-6) B) tumor necrosis factor receptor type 1 (TNFr1) C) D-dimers D) Lipopolysaccharide (LPS) and E) CD14 (sCD14) were measured. Initiation of ART resulted in significant decreases from baseline in plasma levels of IL-6 and TNFr1 by week 4 (p=0.002 and p=0.038) D-dimer levels were significantly reduced by day 7 (p=0.031). Levels of sCD14 were significantly reduced by day 2 following initiation of therapy (p<0.001). LPS levels were significantly reduced from baseline 24 weeks after initiation of ART (p<0.001). None of these markers, except for IL-6, consistently reached the levels seen in healthy controls by the end of the study. Symbols used in the figure: N = Normal controls (in blue). 0 = Baseline. D = Day (The two tick-marks between “0” and “D14” are Day 2 and Day 7). W = Week. * = Change from baseline significantly different from 0 (Wilcoxon signed rank p ≤0.05) . x = Significant difference from the normal controls (Wilcoxon rank sum p ≤0.05). - Horizontal bars represent 25^th^ (Q1), 50^th^ (Median), and 75^th^ percentiles. … Dotted line (in red) connects the medians over time.

Untreated HIV-1 infection is characterized by inflammation and coagulation [27–29]. Plasma levels of IL-6 and D-dimers (products of fibrinolysis) predict mortality in HIV-1 infection [28] and pretreatment plasma levels of the TNFr1 and IL-6 also predicted AIDS events and death in treated patients [30,31]. Initiation of ART decreased plasma levels of IL-6, TNFr1, and D-dimer, by week 4 (p=0.002, p=0.031, and p=0.028, respectively; Figure 3A-3C). Levels of TNFr1 and D-dimers continued to fall throughout the study, but remained higher than among healthy controls. 

We next examined the decay in plasma levels of bacterial LPS and of the soluble LPS coreceptor, sCD14. At weeks 24 and 48, LPS levels were significantly lower than baseline (p< 0.05 for both) yet failed to reach levels seen in uninfected controls (both p<0.001, Figure 3D). Levels of sCD14 decreased rapidly and were significantly lower than baseline by day 2 and continued to fall until they reached a plateau between week 8 and 24; and at weeks 24 and 48 remained higher than levels in uninfected controls (p<0.05 for both; Figure 3E). 

We explored several mechanistic hypotheses by examining correlations among baseline indices (Table 2). We hypothesized that CD8+ T cell cycling was potentially driven by viremia (as we had earlier found that CD8+ T cell cycling was not increased in patients with durable control of viremia [21]) and that CD4+ T cell cycling was likely driven by both viremia and microbial translocation, as CD4+ T cell cycling could be induced in vitro by both bacterial and viral TLR ligands [15]. These hypotheses were not supported by the data, as we did not find a significant association between viremia and CD8+ T cell cycling or between CD4+ T cell cycling and levels of either LPS or sCD14. We did find a positive association between baseline levels of sCD14 and several other markers, including plasma IL-6, D-dimers, TNFr1, and the proportion of both CD4+ and CD8+ T cells that expressed HLA-DR and CD38, suggesting that sCD14, as a marker of monocyte activation/microbial translocation, is related to indices of inflammation, coagulation and T cell activation in HIV-1 disease (Table 2). Soluble CD14 levels at baseline were inversely related to baseline numbers of absolute, naïve, and CM CD4+ T cells, but not to CD8+ T cell counts; consistent with the concept that immune activation/inflammation may drive or be a consequence of CD4+ T cell loss. 

**Table 2:** Correlation coefficients (r) for associations among baseline immunologic indices.

**Baseline outcomes**	***CD4 Count***	***CD8 Count***	***CentralMemoryCD4Count***	***Naïve CD4 Count***	***Naïve CD8 Count***	***% TotalKi67+ CD4+***	***% Total Ki67+ CD8+***	***% Ki67+ Naïve CD4***	***% Ki67+ Central Memory CD4***	***% CD38+/HLA-DR+ CD4***	***% CD38+/HLA-DR+*** ***CD8***	***IL-6***	***TNFr1***	***D-dimer***	***LPS***	***sCD14***	***HIV-1 RNA***
***CD4 Count***																	
***CD8 Count***	0.31																
***CentralMemoryCD4Count***	NA	0.31															
***Naïve CD4 Count***	NA	0.41*	0.56*														
***Naïve CD8 Count***	0.30	NA	0.34*	0.53*													
***% Total***	-0.30	-0.02	-0.28	-0.46*	-0.37*												
***Ki67+ CD4+***																	
***% Total***	-0.19	0.03	-0.07	-0.44*	-0.39*	0.86*											
***Ki67+ CD8+***																	
***% Ki67+***	-0.26	-0.08	-0.18	-0.44*	-0.32	0.88*	0.82*										
***Naïve CD4***																	
***% Ki67+ Central Memory CD4***	-0.44*	-0.05	-0.37*	-0.46*	-0.34*	0.93*	0.78*	0.84*									
***% CD38+/***	-0.60*	-0.23	-0.72*	-0.68*	-0.56*	0.52*	0.38*	0.38*	0.52*								
***HLA-DR+ CD4***																	
***% CD38+/***	-0.20	0.20	-0.22	-0.09	-0.31	0.01	0.06	-0.11	0.03	0.40*							
***HLA-DR+ CD8***																	
***IL-6***	0.05	0.09	0.09	0.15	0.08	-0.02	0.06	-0.08	-0.05	-0.10	-0.04						
***TNFr1***	-0.20	-0.16	-0.26	-0.23	-0.26	0.07	0.13	-0.12	0.002	0.33*	0.26	0.47*					
***D-dimer***	-0.30	0.04	-0.04	-0.22	-0.20	0.18	0.29	0.10	0.25	0.07	0.07	0.41*	0.33*				
***LPS***	0.05	0.32	0.08	0.14	0.21	0.12	0.22	0.08	0.08	-0.14	-0.02	0.005	0.15	0.22			
***sCD14***	-0.35*	-0.006	-0.33*	-0.41*	-0.28	0.24	0.26	0.09	0.16	0.52*	0.47*	0.38*	0.61*	0.38*	0.13		
***HIV-1 RNA***	-0.12	-0.16	-0.15	-0.19	-0.15	0.01	0.01	0.07	0.005	0.07	-0.10	0.26	0.23	0.14	-0.26	0.05	

(Spearman) correlation coefficients are in bold and labeled with an asterisk (*) when the associations are significant (p-value < 0.05).

We next examined the relationship between baseline indices of activation and inflammation and the magnitude of CD4+ T cell restoration. Among the baseline indices examined, levels of CD4+ T cell cycling (r= -0.39, p=0.03), proportions of activated CD38+ HLA-DR+ CD4+ T cells (r= -0.41, p=0.029), and levels of TNFr1(r= -0.43, p=0.013), were correlated negatively with the 48 week increase in total CD4+ T cells. The 48 week increase in naïve CD4+ T cells was correlated negatively with the baseline proportions of cycling CD4+ (r= -0.45, p=0.016), cycling CM CD4+ T cells (r= -0.40, p=0.033), and cycling CD8+ T cells (r= -0.39, p=0.043), and with baseline sCD14 (r= -0.42, p=0.028). No baseline indices significantly correlated with CM CD4+ T cell increases (not shown). As had been shown previously [23], baseline naïve CD4+ T cell counts predicted total CD4+ T cell increases at week 48(r=0.47, p=0.008), as did baseline central memory CD4+ T cell count (r=0.41, p=0.025).

Since different mechanisms are proposed for the biphasic CD4+ T cell restoration, we next asked if any baseline indices of activation are related to either first phase (redistribution) or second phase (expansion) increases. None of the baseline activation, cycling, or inflammatory markers, was correlated with first phase total CD4+ T cell increases (from week 0 to week 4, Table 3). When we examined separately the restoration of phenotypically naïve CD4+ T cells and CM CD4+ T cells, significant correlations were uncovered. Baseline CD4+ (but not CD8+) T cell activation correlated inversely with the magnitude of first phase naïve CD4+ T cell restoration (r= -0.66, p<0.001), as did the baseline proportions of cycling CD4+, naïve CD4+ and CM CD4+ T cells (r= -0.44, -0.42, -0.46, p= 0.007, 0.009, and 0.004, respectively). Among inflammatory indices, baseline levels of sCD14 were inversely correlated with first phase naïve CD4+ T cell restoration (r= -0.43, p=0.008). First phase restoration of CM cells correlated significantly only with baseline levels of IL-6 (r= -0.35, p=0.03). Baseline levels of IL-6 did not predict first phase or second phase restoration of any other maturation subset. Second phase (week 8-48) increases in CD4+ T cells and their naïve and CM subsets, were negatively correlated with baseline proportions of activated CD4+ T cells (r= -0.42, -0.4, and -0.52, p= 0.009, 0.014, and 0.001, respectively), and with their proportions of cycling (r= -0.38, -0.39, and -0.47, p= 0.021, 0.016, and 0.003). Total and CM CD4+ T cell second phase increases correlate negatively with cycling CM CD4+ T cells (r=-0.37 and -0.48, p= 0.022 and 0.003), and negatively with baseline levels of TNFr1 (r= -0.36 and -0.34, p= 0.03 and 0.041). Second phase naïve CD4+ T cell restoration correlated inversely with baseline naïve CD4+ T cell cycling (r= -0.33, p=0.05); but for restoration of total and CM CD4+ T cells, correlations with baseline naïve CD4+ T cell cycling were not significant. The magnitude of baseline HIV-1 viremia was not associated with any indices of CD4+ T cell restoration. 

**Table 3:** Correlation coefficients (r) for associations between CD4 count restoration and baseline immunologic/virologic markers.

**SUBJECT-SPECIFIC ESTIMATE**		**Baseline Markers**									
		***% CD38+/ HLA-DR+ CD4***	***% CD38+/ HLA-DR+ CD8***	***% Total Ki67+ CD4+***	***% Ki67+ Central Memory CD4***	***% Ki67+ Naive CD4+***	***IL-6***	***TNFr1***	***LPS***	***sCD14***	***HIV-1 RNA***
**First-phase restoration**	**CD4 T-cell count**	-0.002	0.19	0.08	0.12	0.05	-0.09	0.15	0.24	0.08	0.16
	**Naïve CD4 count**	-0.66*	-0.14	-0.44*	-0.46*	-0.42*	0.06	-0.19	0.14	-0.43*	-0.09
	**Central Memory CD4 count**	0.14	0.19	0.01	0.05	0.06	-0.35*	-0.01	0.02	-0.14	0.05
***(BL to week 4)***											
**Second-phase restoration** ***(week8toweek48***)	**CD4 T-cell count**	-0.42*	-0.02	-0.38*	-0.37*	-0.23	-0.06	-0.36*	0.17	-0.29	0.02
	**Naïve CD4 count**	-0.40*	0.02	-0.39*	-0.30	-0.33*	0.02	-0.25	0.17	-0.32	-0.06
	**Central Memory CD4 count**	-0.52*	-0.06	-0.47*	-0.48*	-0.28	-0.09	-0.34*	0.17	-0.30	-0.05

(Spearman) correlation coefficients are in bold and labeled with an asterisk (*) when the associations are significant (p-value < 0.05).

We next asked whether decay of immune and inflammatory indices in the first phase (week 0-4) and second phase (week 8-48) were correlated with first (Table 4) or second phase (Table 5) CD4+ T cell restoration. Interestingly, the only significant relationships for first phase cellular restoration were seen for naïve CD4+ T cells, wherein smaller decreases in cycling of all CD4+ T cell subsets were associated with greater CD4+ T cell restoration (Table 4). Smaller second phase decreases in CD4+ T cell cycling and CD4+ T cell subset cycling also significantly correlated with greater second phase naïve CD4+ T cell restoration and also tended to correlate with greater restoration of total CD4+ and CM CD4+ T cells (Table 5). While this may be the consequence of lower baseline cycling, these relationships at baseline and with therapy suggest that in this setting, cycling of CD4+ T cells is less homeostatic than driven by other mechanisms. 

**Table 4:** Correlation coefficients (r) for associations between first-phase cell restoration and immunologic decay.

**SUBJECT-SPECIFIC ESTIMATE**		**First-phase Immunologic Decay (BL to Week 4)**								
		***% CD38+/HLA-DR+ CD4***	***% CD38+/HLA-DR+ CD8***	***% Total Ki67+ CD4+***	***% Ki67+ Central Memory CD4***	***% Ki67+ Naive CD4+***	***IL-6***	***TNFr1***	***LPS***	***sCD14***
**First-phase restoration (*BLtoweek4***)	**CD4 T-cell count**	0.29	0.07	-0.02	0.01	0.003	-0.09	-0.23	-0.19	0.003
	**Naïve CD4 count**	0.11	-0.24	0.57*	0.65*	0.46*	-0.005	0.02	0.09	0.32
	**Central Memory CD4 count**	-0.04	-0.20	-0.08	-0.03	-0.69	0.19	-0.09	0.11	0.06

(Spearman) correlation coefficients are in bold and labeled with an asterisk (*) when the associations are significant (p-value < 0.05).

**Table 5:** Correlation coefficients (r) for associations between second-phase cell restoration and immunologic decay.

**SUBJECT-SPECIFIC ESTIMATE**		**Second-phase Immunologic Decay (Week 8 to Week 48)**								
		***% CD38+/HLA-DR+ CD4***	***% CD38+/HLA-DR+ CD8***	***% TotalKi67+ CD4+***	***% Ki67+ Central Memory CD4***	***% Ki67+ Naive CD4+***	***IL-6***	***TNFr1***	***LPS***	***sCD14***
**Second-phase restoration (*week 8 to week 48*)**	**CD4 T-cell count**	0.10	0.17	0.40*	0.29	0.31	0.19	0.10	0.23	0.32
	**Naïve CD4 count**	0.27	0.18	0.41*	0.34*	0.38*	-0.01	0.22	0.29	0.15
	**Central Memory CD4 count**	0.24	0.29	0.39*	0.39*	0.28	0.22	0.21	0.16	0.40*

(Spearman) correlation coefficients are in bold and labeled with an asterisk (*) when the associations are significant (p-value < 0.05).

## Discussion

The intensive immunologic monitoring in this study allowed fine characterization of treatment related changes in immune and inflammatory indices and an exploration of their relationships to immune restoration. CD8+ T cell numbers increased during the first two weeks, stabilized by week 4, and then fell, such that circulating CD8+ T cell numbers were not different from baseline after 4 weeks. While circulating CD4+ T cell numbers are characteristically low in HIV+ patients, CD8+ T cell numbers are expanded and in this study remained elevated (compared to numbers in healthy controls) throughout 48 weeks.. Typically, HIV-1 reactivity among circulating CD8+ T cells falls with suppression of HIV-1 replication [32,33]. Are these expanded cells reactive with other microbial pathogens (e.g. cytomegalovirus)? Or do they represent long lived cells resistant to cell death? The determinants and significance of this persistent expansion remain to be determined, but does not appear to be a consequence of “blind T cell homeostasis” as a result of CD4+ lymphopenia, as CD8+ T cell expansion is even more demonstrable among ART treated persons who normalize CD4+ T cell numbers than among immune failures who do not [21]. In this study the bulk of CD8+ T cell expansion reflected increases in the numbers of effector memory and terminally differentiated effector memory cells (not shown). 

Confirming previous reports [34], we observed biphasic restoration of CD4+ T cells and their maturation subsets. The determinants of this biphasic cellular restoration are incompletely understood. Earlier work has suggested that the rapid first phase of cellular restoration is most compatible with a redistribution of lymphocytes sequestered in inflamed lymph nodes [13,14] and the second phase is related to increased homeostatic proliferation [35]. As there was no increase in Ki67 expression in any CD4+ T cell population during this time, our data suggest that cellular increases are not a consequence of greater proliferation; but could plausibly be related to less cell death as suggested by DeMascio [35]. In fact, greater CD4+ T cell restoration, in both the first and second phases, was related to lower levels of CD4+ T cell activation and cycling at baseline, and to decreases in these indices following initiation of therapy. Earlier works found an inverse relationship between CD4+ T cell counts and their cycling [35–37], yet increased cycling in HIV infection could reflect either homeostatic responses to cytopenia, or may drive cell losses as these cells die, or both. The data generated in this prospective clinical trial of antiretroviral therapy are compatible with a role for CD4+ T cell cycling in pathogenesis of cell losses. In this regard, baseline levels of inflammatory markers, TNFr1, IL-6, and sCD14, were negatively associated with increases in CD4+T cell populations. We have recently found that the inflammatory cytokines IL-6 and IL-1β drive both CD4 T cell turnover and diminish CD4 T cell responses to the homeostatic cytokine IL-7 [38]. 

A growing body of evidence links immune activation and inflammation to the pathogenesis of immune deficiency [2,3,9,10,21] and to morbid outcomes of HIV-1 infection [28]. Here, both CD4+ and CD8+ T cells remained activated throughout the course of this study, and although the proportions of activated T cells diminished, they remained significantly higher than among healthy controls. In fact, in other studies, activation of CD4+ and CD8+ T cells can persist despite years of virologic control [3,21], and we found persistent activation despite declining viremia below 50 copies of HIV-1 RNA/mL as measured by single copy assay [19]. While the determinants of persistent activation in this setting remain incompletely understood, the data here implicate activation and inflammation also in the pathogenesis of immune deficiency. 

A unique feature of our study was the frequent measurement of immune activation markers soon after the initiation of ART. Notably, several markers of immune activation and inflammation began to normalize within days of starting ART.  We found no significant relationship between the early changes in plasma HIV-1 RNA level and changes in activation/inflammation markers or cellular restoration. This may result from the exclusion from the trial of subjects with low levels of viral replication (<10,000 copies/mL) and the relatively small size of the study. 

 And despite rapid reduction in plasma HIV-1 RNA levels, systemic levels of LPS and its soluble coreceptor, sCD14 remained elevated for the duration of the study. These results suggest that factors other than ongoing viral replication may provide the stimulus to persistent immune activation in HIV-infected patients. These observations are consistent with earlier observations that in most instances the damage to gut immunity does not completely resolve with ART [39]. It is also clear that sCD14 decays much more rapidly from plasma than does LPS, consistent with the concept that factors other than LPS also may drive plasma levels of this coreceptor [40]. 

Recent works have linked the morbidities and mortalities of treated HIV-1 infection to increased inflammatory and coagulation indices [28,31,41]. Here, levels of the inflammatory cytokine IL-6, the soluble cytokine receptor TNFr1, the LPS coreceptor sCD14, and D-dimer markers of fibrinolysis, fell during ART; yet, despite one year of effective therapy, plasma levels of TNFr1, sCD14, and D-dimers, remained higher than among healthy controls. 

By examining the relationships between activation/inflammation indices and both first phase and second phase cellular restoration, we have found that both first phase and second phase naïve CD4+ T cell restoration was linked inversely to the magnitude of first and second phase decreases in CD4+ T cell cycling and activation. Though less robust, the second phase recovery of all CD4+ T cells and of CM CD4+ T cells was also linked inversely to the decrease in CD4+ T cell cycling. These data suggest that while T cell activation and inflammatory/coagulation markers may be linked to immune deficiency and HIV-related morbidity and mortality, the factors that drive them may be complex and distinguishable in terms of their relationship to immune restoration. Our data are more compatible with a model wherein activation and cycling are driving pathogenesis rather than being reflective of homeostasis. While the processes that drive immune deficiency and inflammation are triggered by HIV-1 infection, suppression of HIV-1 replication with effective ART does not fully correct the residual immune dysregulation that even after a year of therapy is linked to the newer morbidities of HIV-1 infection. 

## Supporting Information

Checklist S1 **CONSORT Checklist.**
(DOC)

Protocol S1 **Trial Protocol.**
(DOC)
